# sTREM-1 as a Prognostic Marker of Postoperative Complications in Cardiac Surgery

**DOI:** 10.5402/2012/382862

**Published:** 2012-12-17

**Authors:** A. S. Golovkin, V. G. Matveeva, E. V. Grigoriev, D. L. Shukevich, Y. V. Bayrakova, L. S. Barbarash

**Affiliations:** Research Institute for Complex Issues of Cardiovascular Diseases, The Siberian Branch of The Russian Academy of Medical Sciences, 650002 Kemerovo, Russia

## Abstract

Cell-activating receptor TREM-1 (triggering receptor expressed on myeloid cells 1) regulates congenital immune response and contributes to systemic inflammatory response syndrome (SIRS) development. It is able to multiply cytokine production while stimulated together with the main receptors of the congenital immune system. The purpose of the paper is to study the potential use of soluble TREM-1 (sTREM-1) as a marker of intensive SIRS and a criterion for postoperative complications prediction following on-pump coronary artery bypass surgery (CABG). Results show that early postoperative sTREM-1 concentrations demonstrate their potential prognostic value regarding SIRS-associated complications.

## 1. Introduction

 On-pump open-heart surgery results in the development of systemic inflammatory response syndrome (SIRS) of various intensity. Severe SIRS complications are multiple organ failure (MOF), acute respiratory distress syndrome (ARDS), or shock. SIRS diagnosis criteria, approved in 1991 at the ACCP/SCCM Consensus Conference in Chicago, have been reasonably criticized for their nonspecificity and low sensitivity [1]. SIRS can be diagnosed if two or more criteria are present:

- body temperature >38°C or <36° C;
- heart rate >90 bpm;
- respiratory rate >20 breaths/min;
- white blood cell count >12*10^9^/L or <4*10^9^/L;
- proportion of young granulocytes >10%.

The drawbacks of these criteria are “classical”: too high sensitivity, no specificity for infectious and noninfectious etiology of the disease and critical condition, and the fact that they are difficult to effectively observe because of all intensive care procedures. Therefore, there are almost no effective and specific criteria for laboratory SIRS diagnosis, especially in patients after on-pump open-heart surgeries.

An important pathogenetic step in developing secondary systemic injury and forming a “vicious circle” in SIRS is early inflammatory activation. As a result, high concentrations of cytokines, proteinases, and reactive oxygen species (ROS), which are mainly produced by monocytes, neutrophils, tissue, and vascular macrophages, are released into the blood [2]. 

Triggering receptor expressed on myeloid cells-1 (TREM-1) has been considered to play an important role in early inflammation activation [3]. It was first described as inflammation activating receptor in 2000 by a group of Swiss researchers, that is, A. Bouchon, J. Dietrich *и* Colonna M. J. A characteristic feature of TREM-1, which makes it attractive for SIRS research, is its ability to activate all effector functions of the congenital immune system and multiply cytokine production if stimulated together with the main receptors of the congenital immune system (toll-like receptors and Nod-like receptors) [4]. Until recently, it has been considered a marker of sepsis and noninfectious SIRS. However, in the last years there appeared some research data that suggested the association between TREM-1 and noninfectious SIRS [5–7].

Soluble TREM-1 (sTREM-1) is released from the surface when the membranous form is shedd by matrix metalloproteinases and can be quantitatively measured in biological substances. 

 Considering that this receptor is capable of amplifying the inflammatory response and some new research data on this receptor participation in the development of noninfectious SIRS a study investigating the potential use of soluble TREM-1 as a marker of postoperative SIRS intensity and its complications after direct on-pump myocardial revascularization can be of interest [8, 9].

*Purpose*: is investigate the potential use of soluble TREM-1 (sTREM-1) as a marker of SIRS intensity and criterion for early postoperative complications prediction after on-pump coronary artery bypass surgery (CABG). 

## 2. Materials and Methods

67 patients with ischemic heart disease, angina pectoris II-III CCSC, chronic heart failure (CHF) NYHA II-III, and aged 50–70 years, were enrolled in the study. The exclusion criteria were concomitant coronary and valvular diseases, acute infection and chronic infection exacerbation, malignancies, or postoperative surgical complications. 

Baseline left ventricular ejection fraction was >40%. All the patients had CABG performed with standard cardioplegia and nonpulsatile cardiopulmonary bypass (CPB). Intravenous general anesthesia with fentanyl and midazolam was introduced. Homotypic hypertonic hyperoncotic perfusate was used for CPB with the same initial amount in the heart-lung machine. Cold blood cardioplegia was used, and cardioplegia passage was done anterogradely. The bypass time was 88 min (75–105 min), and aortic cross-clamp time was 57 min (48–61 min). Peripheral venous blood was drawn into the clot activator tubes before the surgery, 18 hours and 7 days after the surgery. 

In order to dynamically assess the severity of organ failure before the surgery and 1 day after the surgery (Sepsis SOFA-related Organ Failure Assessments Score/Sequential Organ Failure Assessment) score was used. The overall SOFA score consists of separate scores indicating the severity of different system failures (respiratory, cardiovascular, nervous, coagulation, hepatic, and renal). 

Serum soluble TREM-1 (Human TREM-1) concentrations were measured by ELISA with R&D systems (USA), interleukin-6 (IL-6) (Human IL-6) and C-reactive protein (CRP) (Human CRP ELISA) levels were measured with Bender MedSystems (USA).

Statistical data processing was done in “Statistica 6.0” software. The significance of the differences was defined with non-parametric Wilcoxon-Mann-Whitney *U*-test and Wilcoxon *W*-test. The groups were compared with ANOVA. The differences were considered to be statistically significant if *P* < 0.05. The data are presented as a median and an interquartile range (25–75%).

## 3. Results

### 3.1. General Characteristics of the Patients

All the patients developed SIRS with more than 2 criteria at day 1 after the surgery. The average SOFA scores demonstrating the severity of multiple organ failure were 2.5 (1–6 scores). 

On day 1 after the surgery serum IL-6 and CRP concentrations were 4-fold and 16-fold higher than the preoperative values, respectively (Table 1). An reasonable response to operative stress was a release of inflammation mediators. By day 7 after the surgery blood IL-6 concentrations did not differ from the preoperative values. CRP increase was more stable. Serum CRP concentrations significantly decreased by day 7 but were still higher than the preoperative values. 

Serum soluble TREM-1 (sTREM-1) dynamics was different. After their significant rise at day 1 sTREM-1 levels kept increasing up to day 7. 

### 3.2. Patient Grouping

According to the postoperative course all the patients were divided into 3 groups. Group 1 included those with minimal SIRS at day 1 and SOFA scores 1–3 (*n* = 57). Group 2 included those with MOF-complicated SIRS (day 1 SOFA scores 6.2 (4–8)) (*n* = 5). Group III had patients with renal dysfunction developed at days 1–3 after the surgery (defined by the decreased eGFR, hyperazotemia) (*n* = 5). 

### 3.3. sTREM-1 Levels in Different Patient Groups

ANOVA showed that the groups of patients under study did not differ by baseline blood sTREM-1 concentrations (Table 2). 

18 hours after the surgery all the groups were observed to have increased sTREM-1 levels. Patients with complicated SIRS had significantly higher sTREM-1 concentrations than those in the other groups (*P* = 0.028).

At day 7 after the surgery there were no significant differences in sTREM-1 levels; however, there was a tendency towards higher levels in patients with complications. 

### 3.4. Rationale for the Use of “Increase Rate”

The analysis showed that absolute sTREM-1 values did not demonstrate real changes due to significant baseline differences. In order to eliminate this drawback the value of sTREM-1 concentration increase rate was introduced based on the following formula:

(1) Rate=concentration  at  the  time  of  the  studybaseline  (preoperative)  concentration.

### 3.5. The Increase Rate in the Study Groups

**Figure 1:**
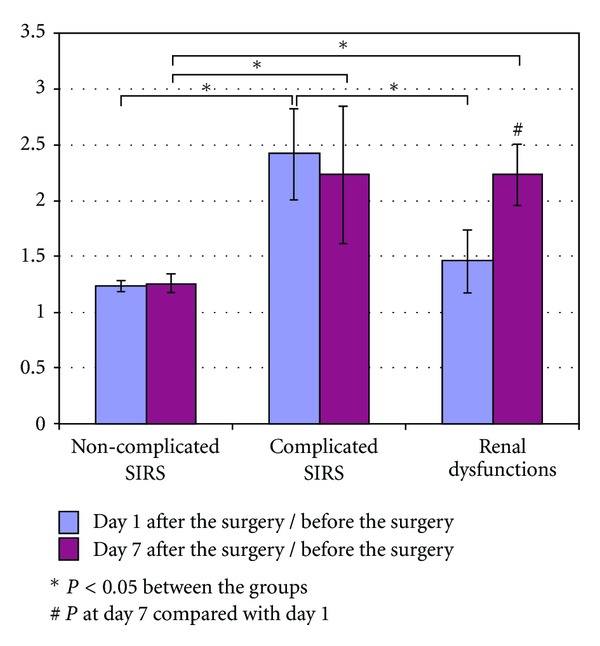
Dynamics of serum sTREM-1 increase rate in the different patient groups.

The coefficient was calculated and compared in the groups using ANOVA. It was found that day 1 after the surgery patients who were going to develop complicated SIRS had significantly higher increase rate (1.68 (1.56–2.47)) compared with the groups having normal postoperative period (1.19 (0.98–1.36), *P* < 0.001) and those with renal dysfunction (Figure 1).

At day 7 after the surgery the increase rate was significantly higher in those with complicated SIRS (1.76 (1.72–3.76)) and renal dysfunctions (2.38 (2.24–2.70)) compared with the group where there were no complications (1.29 (1.03–1.46)) (*P* < 0.001 and *P* < 0.001, resp.).

Thus, in noncomplicated SIRS the increase rate was relatively stable at days 1 and 7 (1.19–1.29), respectively, being insignificant. 18 hours after the surgery there was a 1.5-fold rise in the increase rate in patients with complicated SIRS. 

Patients with renal dysfunctions had this parameter increased up to 2.38 by day 7. 

Postoperative sTREM-1 increase dynamics can effectively demonstrate the development of a complicated systemic inflammatory response and multiple organ failure.

**Clinical Case 1**

Patient I, 58 years old, medical records number 5644, was diagnosed with polyvascular disease, ischemic heart disease, angina pectoris CCSC II, right common carotid artery stenosis of 30% and arterial hypertension III, risk 4. Preoperative sTREM-1 concentration was 97.11 pg/mL. Complete on-pump myocardial revascularization was done on August 31, 2010 (1 mammary and 2 venous grafts). The bypass time was 85 min, aortic cross-clamp time 64 min, and operation time 300 min. He was transferred to the ICU with stable haemodynamics and had no sympathomimetic support. He was extubated 2.5 hours after the transfer with blood gases being adequate. Early after the surgery the condition was stable and consistent with the severity of the performed procedure. On the next day the body temperature ranged from 36.1 to 36.7; the heart rate was >82 bpm and the respiratory rate was not more than 18 breaths/min with arterial   PaCO_2_ not less than 34 mmHg in spontaneous breathing. Peripheral blood was as following: white blood cell count 9.2*109 and young granulocytes 5%.

The standard diagnosis criteria showed noncomplicated SIRS. sTREM-1 level was 75.96 pg/mL. The rate of sTREM-1 increase was 0.78. Noncomplicated SIRS was confirmed. Noncomplicated postoperative follow-up period was predicted. The patient was discharged form the ICU in a stable condition on September 01, 2010. Discharged from hospital in a stable condition on September 10, 2010.

**Clinical Case 2**

Patient L, 68 years old, medical records numbers 4493, was diagnosed with polyvascular disease, ischemic heart disease, previous myocardial infarction (2007), angina pectoris CCSC II, right common carotid artery stenosis of 40%, and arterial hypertension III, risk 4. Preoperative sTREM-1 concentrations were 54.71 pg/mL. Complete on-pump myocardial revascularization was done on July 07, 2010 (1 mammary and 2 venous grafts). The bypass time was 93 min, aortic cross-clamp time 69 min, and operation time 285 min. He was transferred to the ICU with stable haemodynamics and no sympathomimetic support. He was extubated 3 hours after the transfer with blood gases being adequate. Early after the surgery the condition was stable and consistent with the severity of the performed procedure. 8 hours after the surgery a progression of vasoplegia occurred requiring adrenaline infusion to support adequate mean blood pressure. Due to the worsening of arterial and venous blood gas content the patient was reintubated and placed on a ventilatory support. Lower urine output was observed, which required stimulation with furosemide and hypovolemia treatment. A complicated SIRS was suspected. However, white cell count was 10.4*109, young granulocytes 8%, postoperative body temperature ranged from 36.0 to 37.2, and the heart rate was >86 bpm. The respiratory rate and arterial blood   PaCO_2_ were insignificant as diagnostic criteria because the patient was ventilated. sTREM-1 level was 135.10 pg/mL. The rate of sTREM-1 increase was 2.47. The diagnosis of a complicated SIRS was confirmed. The patient's condition got worse during two days due to the development and progression of multiple organ failure. At day 7 after the surgery the rate of sTREM-1 increase was 4.56. For extracorporeal blood purification prolonged venovenous hemofiltration was done, which resulted in the regression of multiple organ failure clinical and laboratory signs. The patient was discharged from the ICU on July 12, 2010.

## 4. Discussion

Early after the surgery patients who have undergone on-pump open-heart surgery and develop systemic inflammatory response of various intensity with concurrent hypercytokinemia. We chose IL-6 and CRP as SIRS indicators. IL-6 is an early mediator and has a longer half-life period than TNF*α* and IL-1*β* and that intrinsic property makes it possible to use IL-6 as a marker of sepsis and SIRS [2, 10, 11]. Proinflammatory cytokines (IL-1, IL-6, and TNF*α*) induce the production of acute phase C-reactive protein in the liver, of which level is characteristic of the inflammatory response intensity. In our study post-CABG SIRS was confirmed by the presence of the approved diagnosis criteria and multiplefold increase in IL-6 and CRP. 

TREM-1 contributes to the activation of congenital immune system effector functions [3, 12]. It was demonstrated before that serum sTREM-1 level correlated with the severity of sepsis and represented a highly sensitive (96%) and specific (89%) marker of sepsis [12, 13]. However, recent clinical trials indicate that sTREM-1 increases in patients with nonspecifically activated SIRS after surgeries involving cardiopulmonary bypass, cardioplegia, resuscitation, blood loss, and hemotransfusion [14]. The increase in surface TREM-1 expression on monocytes and serum sTREM-1 concentrations was registered in patients with postoperative preclinical SIRS and no sepsis [5], in those with acute pancreatitis [6] and pulmonary contusion [7]. In these conditions the severity of the process correlates with the level of TREM-1 expression on monocytes. Consequently, in this patient category TREM-1 can not be used as a marker of infectious complications but can serve as an indicator of inflammation intensity. 

It was found that soluble TREM-1 is formed in a human body as a result of TREM-1 membrane shredding (proteolytic detachment) from the surface of myeloid cells by matrix metalloproteinases (MMP) [13]. Therefore, the increase in blood sTREM-1 level indirectly reflects the increase in membranous TREM-1 expression in the early inflammation phase. 

In our study, registered serum sTREM-1 increase in patients, who have undergone on-pump open-heart surgery with cardioplegia, is in accordance with Adib-Conquy's suggestion that the increase in TREM-1 mostly reflects systemic inflammation more than an infectious process [14]. The patients examined in our study did not have any postoperative septic complications but all of them had SIRS signs of various intensity. 

The analysis of our results and literature data showed that blood sTREM-1 concentrations were very individual. The use of sTREM-1 increase rate allows to follow its dynamics at different postoperative time points compared with the preoperative values. It was found that 18 hours after the surgery the group, which then developed complicated SIRS, had significantly different sTREM-1 levels. This indicates pathogenetic significance of sTREM-1 involvement into SIRS and its complicated forms of development. 

The literature contains mechanisms and ways of sTREM-1 elimination. The increase in blood sTREM-1 concentrations in patients with renal dysfunctions observed in our study allows to suggest the involvement of the kidneys into sTREM-1 elimination. 

The abovementioned clinical cases demonstrate diagnostic and prognostic value of postoperative sTREM-1 dynamic assessment following on-pump CABG. Indeed, both patients had comparable extent of surgery, bypass time, aortic cross-clamp time, and the duration of surgery. At the same time, despite progressive worsening of the patient's condition in case 2 early after the surgery due to SIRS and MOF the analysis of common SIRS diagnosis criteria did not allow prompt diagnosis of the condition. sTREM-1 increase rate at day 1 and day 7 after the surgery was 2.47 and 4.56, respectively, which indicated not only SIRS development, but also its complicated course, that is, multiple organ failure. 

Thus, more than 1.5-fold increase in sTREM-1 concentrations at day 1 after the surgery can indicate the intensity of systemic inflammatory response and possible associated complications. High increase rates later after the surgery are associated with complicated SIRS and, moreover, correlate with renal dysfunction. 

Early postoperative sTREM-1 levels demonstrate their potential prognostic and diagnostic value in regards to SIRS-associated complications.

## Figures and Tables

**Table 1:** Dynamics of the parameters under study in patients who have undergone on-pump CABG; median (Q25–Q75).

Parameter	Before the surgery	Day 1 after the surgery	Day 7 after the surgery
IL6 pg/mL	2.12 (1.05–2.47)	8.75 (3.32–13.47)*	2.64 (1.78–3.63)**
CRP mg/L	2.71 (1.50–4.66)	44.55 (37.97–49.13)*	35.60 (25.60–45.78)^∗/∗∗^
sTREM-1 pg/mL	58.75 (46.71–104.20)	80.60 (60.30–119.1)*	125.10 (72.17–165.90)^∗/∗∗^

*P < 0.05 compared with the preoperative levels.

**P < 0.05 compared with Day 1 levels.

**Table 2:** Dynamics of serum sTREM-1 (pg/mL) in the different patient groups; median (Q25–Q75).

Groups	Before the surgery	Day 1 after the surgery	Day 7 after the surgery
Non-complicated	58.06	74.98*	101.20*
SIRS (*n* = 57)	(46.53–109.20)	(58.99–107.9)	(68.45–162.55)
Complicated SIRS	67.46	131.10^∗/#^	157.50*
(*n* = 5)	(54.71–77.90)	(130.50–135.10)	(134.00–249.30)
Renal dysfunctions	64.50	98.16	150.95*
(*n* = 5)	(57.42–82.76)	(79.10–133.45)	(105.36–183.95)

*P < 0.05 compared with the preoperative levels.

^#^ *P* < 0.03 compared with the level of the same parameter in the non-complicated SIRS group.
